# Efficacy of transumbilical single-port and two-port laparoscopy in the treatment of pediatric inguinal hernia: a systematic review and meta-analysis

**DOI:** 10.3389/fped.2026.1814850

**Published:** 2026-05-08

**Authors:** Bo Yang, Yi Zhou, Shipeng Tang

**Affiliations:** Department of Gastrointestinal Surgery, The People’s Hospital of Leshan, Leshan, Sichuan, China

**Keywords:** inguina, inguinal hernia, pediatric, single-port laparoscopy, two-port laparoscopy

## Abstract

**Objective:**

To systematically evaluate the perioperative efficacy and safety of transumbilical single-port laparoscopic surgery and two-port laparoscopic surgery in the treatment of pediatric inguinal hernia, and to provide high-quality evidence-based medical evidence for the selection of clinical surgical methods.

**Methods:**

A comprehensive search was conducted in PubMed, Embase, Cochrane Library, and Web of Science databases to collect relevant controlled studies published from the establishment of the databases to December 31, 2025. After screening the literature and extracting data according to the pre-set inclusion and exclusion criteria, the ROB2 tool and ROBINS-I tool were used to evaluate the risk of bias in RCT and non-RCT studies, respectively. Meta-analysis was performed using RevMan 5.3 software and R software.

**Results:**

A total of 13 studies were included, involving 22,846 children. Meta-analysis showed that the postoperative recurrence rate in the single-port laparoscopic group was significantly lower than that in the two-port laparoscopic group (RR = 0.60, 95% CI: 0.39–0.94, *P* = 0.02); there were no statistically significant differences between the two groups in terms of operation time (MD = −1.43, 95% CI: −3.42–0.57, *P* = 0.16), hospital stay (MD = −2.34, 95% CI: −7.73–3.06, *P* = 0.40), detection rate of contralateral occult hernia (RR = 1.03, 95% CI: 0.94–1.13, *P* = 0.55), conversion to open surgery rate (RR = 1.57, 95% CI: 0.14–17.93, *P* = 0.71), and the incidence of various postoperative complications (all *P* > 0.05). Subgroup analysis showed that in large sample size studies, the operation time in the single-port laparoscopic group was significantly shorter (MD = −4.27, 95% CI: −7.25–1.28, *P* < 0.001), and in small sample size studies, the recurrence rate advantage of the single-port laparoscopic group was more significant (RR = 0.43, 95% CI: 0.25–0.71, *P* = 0.001).

**Conclusion:**

Transumbilical single-port laparoscopic surgery has a significant advantage in reducing postoperative recurrence rate in the treatment of pediatric inguinal hernia. In large sample size studies, its advantage in operation time is more evident, and its safety is comparable to that of two-port laparoscopic surgery. For medical centers with mature single-port operation techniques, single-port laparoscopy can be the preferred surgical method; for complex hernias or when technical conditions are limited, two-port laparoscopy remains a reliable choice.

## Introduction

Pediatric inguinal hernia is one of the most common diseases in pediatric surgery worldwide, with an incidence rate of approximately 1%–5% in the neonatal period, and over 80% of the affected children are male. The incidence of bilateral hernias is about 10%–15% (1). The core mechanism is the failure of the congenital peritoneal processus vaginalis to close (2). Under the induction of factors that increase intra-abdominal pressure, such as crying, coughing, and constipation, the intestinal contents and omentum in the abdominal cavity protrude through the unsealed processus vaginalis to form a hernia sac (3, 4). If not intervened in time, it may lead to serious complications such as incarceration of the hernia contents, intestinal necrosis, and testicular ischemic atrophy, even threatening life (5). Therefore, surgical treatment is the only effective way to cure pediatric inguinal hernia (6).

Open high ligation of the hernia sac was once considered the gold standard for treating pediatric inguinal hernia (7). However, with the rapid development of laparoscopic technology, laparoscopic high ligation of pediatric inguinal hernia has more obvious advantages over traditional open surgery, such as less trauma, better cosmetic effect, lower recurrence rate, and fewer postoperative complications (8, 9). The traditional laparoscopic surgical approach is multi-port or two-port surgery. With the shortening of the learning curve for laparoscopic surgery among surgeons and the pursuit of minimally invasive surgery by people, single-port laparoscopy has gradually been used to treat pediatric inguinal hernia (10). Single-port laparoscopy usually inserts the laparoscope and instruments through a single incision in the umbilicus, resulting in almost invisible scars and reducing the risk of injury related to multiple punctures and postoperative pain (11). However, single-port surgery has greater limitations in terms of suture angles and operating space, leading to problems such as instrument interference and increased surgical difficulty (12). This requires the operator to have advanced laparoscopic skills. In addition, some studies have raised questions about the recurrence rate and surgical safety of single-port laparoscopy when the internal ring is large or multiple peritoneal folds exist (13). In contrast, multi-port laparoscopy provides more space for surgery and more direct suturing. However, it increases the number of abdominal wall incisions, which may affect the cosmetic effect and increase the risk of complications (14).

Currently, most studies on the treatment of pediatric inguinal hernia with single-port and two-port laparoscopy are retrospective analyses and single-center randomized controlled studies. These studies often have deficiencies due to insufficient sample size and inconsistent designs, resulting in a lack of systematic and objective evidence to help clinicians evaluate and choose the best approach. Although there are meta-analyses comparing the advantages and disadvantages of single-port and multi-port approaches for repairing pediatric inguinal hernia (15), the evidence-based medical evidence for the treatment of pediatric inguinal hernia with single-port and two-port laparoscopy is still insufficient.

Therefore, this study aims to integrate the published research results through systematic review and meta-analysis to explore the differences in safety and effectiveness between single-port and two-port laparoscopic techniques in pediatric inguinal hernia repair. It is hoped that this will provide a reference for the clinical promotion and application of single-port and two-port laparoscopy in the treatment of pediatric inguinal hernia.

## Methodology

### Research design and registration

This study is a systematic review and meta-analysis aimed at comparing the perioperative efficacy and safety of umbilical single-port laparoscopic surgery and double-port laparoscopic surgery in the treatment of pediatric inguinal hernia. The research design strictly follows the Preferred Reporting Items for Systematic Reviews and Meta-Analyses guidelines (16) to ensure transparency and reproducibility of the process. The research protocol has been registered on the International Prospective Register of Systematic Reviews (PROSPERO) platform (registration number: CRD420261304172), and all analysis procedures comply with the pre-defined protocol without any substantive modifications.

### Literature search strategy

A comprehensive search was conducted in PubMed, Embase, Cochrane Library, and Web of Science databases, with the search period ranging from the establishment of each database to December 31, 2025. The search was carried out using a combination of subject terms and free words, with key search terms including: Inguinal hernia, Single-port laparoscopic, Double-port laparoscopic, Children, Pediatric, etc. Additionally, the reference lists of the included studies were manually searched to avoid missing potentially eligible studies. The detailed search strategies for each database are presented in Supplementary Table S1.

### Inclusion and exclusion criteria for literature

Inclusion Criteria: 1. The research subjects are children with inguinal hernia aged ≤18 years; 2. The intervention measures are transumbilical single-port laparoscopic surgery, while the control measures are two-port laparoscopic surgery; 3. The research type is a randomized controlled trial (RCT) or non-randomized controlled clinical study; 4. At least one of the following outcome indicators is reported: operation time, hospital stay, postoperative complications (including hernia recurrence, hydrocele, incision infection, etc.), detection rate of contralateral indirect hernia, conversion to open surgery rate.

Exclusion Criteria: 1. Non-controlled studies, case reports, reviews, conference abstracts; 2. Incomplete data or data that cannot be extracted; 3. Republished literature; 4. Research subjects undergoing other concurrent laparoscopic surgeries.

### Literature screening and data extraction

Two researchers independently retrieved the literature according to the search strategy. Firstly, they initially excluded the papers that clearly did not meet the inclusion criteria by reading the titles and abstracts. Then, they obtained the full texts of the potentially eligible papers and conducted the final screening based on the inclusion and exclusion criteria. In case of disagreement during the screening process, they resolved it through discussion between the two researchers; if no agreement was reached, a third senior researcher made the decision.

A standardized data extraction form was used to extract the information, including the first author, publication year, country, sample size, age of the children, gender distribution, type of hernia (unilateral/bilateral), surgical details, outcome indicators, and follow-up time. The primary outcome indicators were the operation time and the incidence of hernia recurrence; the secondary outcomes included the detection rate of contralateral hernia, the rate of conversion to open surgery, postoperative complications, and hospital stay. After extraction, cross-checking was conducted to ensure accuracy.

### Literature quality evaluation

The Cochrane Risk of Bias 2 (ROB2) tool (17) was used to assess the risk of bias in RCTs. This assessment mainly covered 5 core evaluation areas, namely the randomization process, deviation from the expected intervention, missing outcome data, outcome measurement, and reporting bias. Each area was rated at three levels: “low risk of bias”, “some concerns exist”, and “high risk of bias”. The overall bias risk level of the study was determined based on the comprehensive assessment of these areas.

The Risk of Bias in Non-randomized Studies of Interventions (ROBINS-I) tool (18) was used to evaluate non-randomized controlled studies. The evaluation was mainly conducted from 7 dimensions: confounding factors, participant selection, intervention classification, deviation from the expected intervention, missing outcome data, outcome measurement, and reporting bias. Each dimension was rated at four levels: “low risk of bias”, “moderate risk of bias”, “severe risk of bias”, and “extremely severe risk of bias”. The overall bias risk of the study was determined based on the comprehensive assessment of these dimensions.

### Statistical analysis

Statistical analysis was conducted using RevMan 5.3 software and R software. All statistical analyses were performed using the random effects model to fully consider the possible clinical and methodological heterogeneity among the studies. Continuous variables (operation time, hospital stay) were presented as mean difference and 95% confidence interval as the effect size. Binary variables (recurrence rate, complication occurrence rate) were presented as risk ratio and 95% confidence interval as the effect size. Heterogeneity among studies was evaluated using the I^2^ statistic: I^2^ ≤ 50% indicated acceptable heterogeneity, and I^2^ > 50% suggested significant heterogeneity. Grouping analysis was conducted based on factors that might lead to heterogeneity. The preset subgroups included: study type (RCTs vs. non-RCT studies), sample size (less than 500 cases and more than 500 cases), and follow-up time (less than 12 months and more than 12 months). Sensitivity analysis was performed by eliminating individual studies one by one to observe whether the combined effect size changed significantly, in order to verify the robustness of the results. When the number of included studies was ≥5, the publication bias was intuitively judged through the funnel plot; at the same time, Egger's regression test was used for quantitative analysis. If *P* > 0.05, it indicated no significant publication bias. The significance level was set at *α* = 0.05.

## Result

### Literature screening process and results

**Figure 1:**
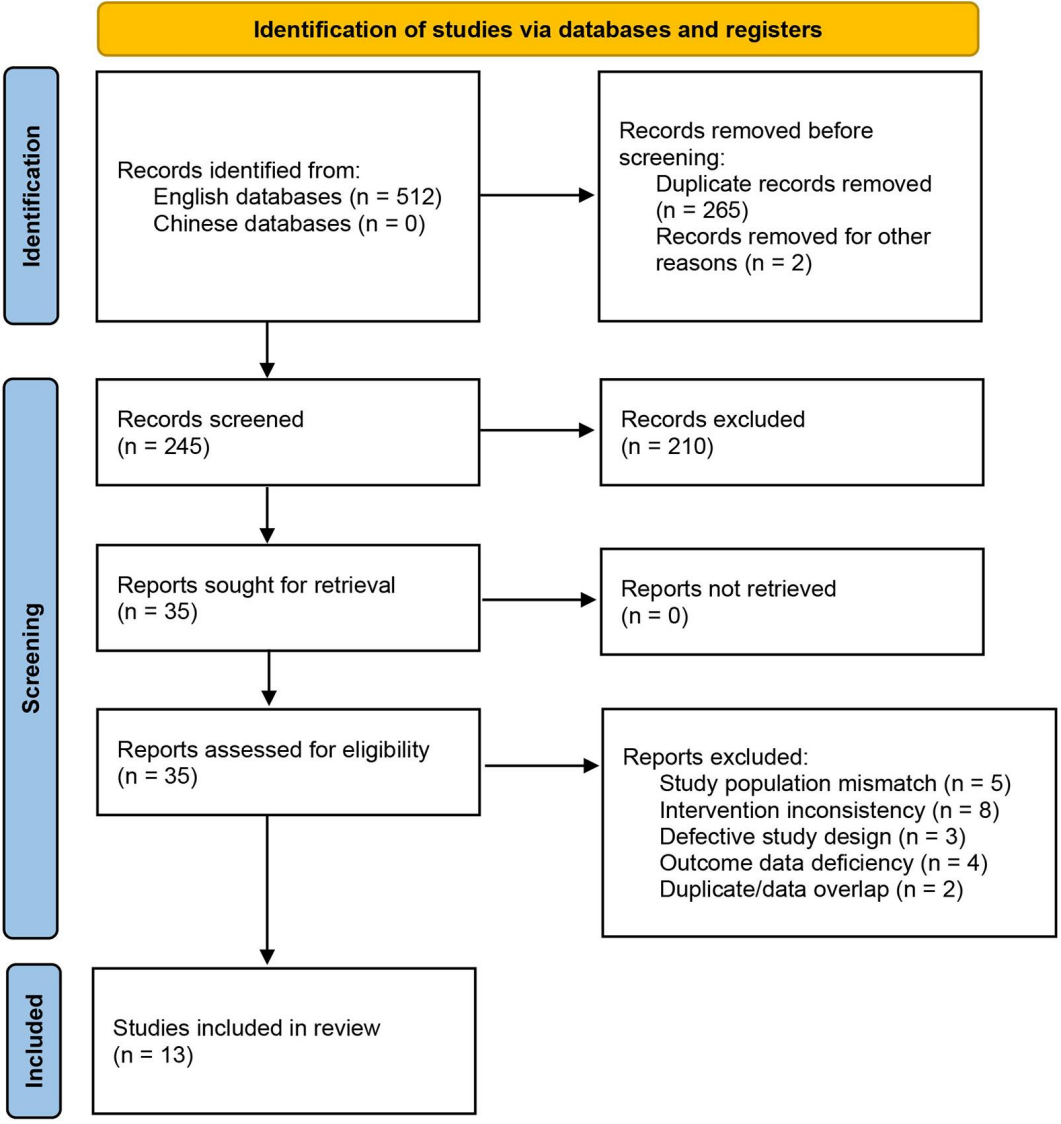
Flow diagram of studies included in meta-analysis.

A total of 512 relevant articles were initially retrieved. After removing duplicates using EndNote literature management software, 245 articles were obtained. Through initial screening based on titles and abstracts, 210 obviously irrelevant articles were excluded, leaving 35 articles for full-text reading assessment. Finally, 13 articles that met the preset inclusion and exclusion criteria were included in this study, including 2 randomized controlled trials (RCTs) (19, 20) and 11 cohort studies (11, 21–30). The literature screening process is shown in Figure 1 (PRISMA flowchart).

### Basic characteristics of the included studies

The total number of children included in the studies was 22,846. Among them, 11,653 cases were in the group that underwent transumbilical single-port laparoscopic surgery, and 11,193 cases were in the group that underwent traditional two-port laparoscopic surgery. The publication time span of the included studies ranged from 2010 to 2025, and the sample size ranged from 109 to 11,298 cases. All studies clearly reported the surgical methods, outcome indicators, and follow-up data. The follow-up duration was from 1 month to 5 years. The baseline data of the included studies showed that there were no significant differences in patient age, gender composition, and distribution of hernia types (unilateral hernia, bilateral hernia) between the single-port group and the two-port group. The baseline data of the two groups were comparable (Table 1).

**Table 1:** Baseline characteristics of included studies for meta-analysis.

Research (Author/Year)	Region	Research type	Sample size (*n*)		Age (years)		Sex (M/F)		Hernia type (unilateral/bilateral)		Follow-up time (months)
			Single-port group	Double-port group	Single-port group	Double-port group	Single-port group	Double-port group	Single-port group	Double-port group	
Uchida 2010	Japan	Retrospective	60	117	4.26 ± 2.92	4.83 ± 2.96	31/29	50/67	39/21	71/46	6
Kozlov 2015	Russia	Retrospective	180	80	0.13 ± 0.06	0.15 ± 0.06	128/52	59/21	116/64	53/27	≥6
Obata 2015	Japan	RCT	37	72	4.55 ± 2.74	4.19 ± 2.68	0/37	0/72	16/21	39/33	12
Peng 2016	China	Retrospective	193	203	1.89 ± 0.38	1.91 ± 0.40	193/0	203/0	142/13	181/22	36–108
Cao 2018	China	Retrospective + PSM	135	135	2.8 ± 1.24	2.7 ± 1.32	85/50	81/54	99/36	102/33	1–24
Wang 2018	China	Retrospective	508	502	4.32 ± 1.62	4.46 ± 1.34	440/68	452/50	NA	NA	24
Luo2022	China	Retrospective	3,339	2,367	2.62 ± 1.38		3,056/283	1,972/212	2,513/826	1,731/453	54 (12–93)
Yi 2022	China	Retrospective	190	99	2.54 ± 2.05	2.45 ± 1.82	168/22	81/18	92/ 88	55/ 44	9.8 ± 3.4
											9.6 ± 3.2
Liu 2023	China	Retrospective + PSM	465	465	2.98 ± 2.34	3.04 ± 1.36	386/79	384/81	354/111	351/114	32.2 ± 7.3
											32.3 ± 9.0
Li 2024	China	RCT	255	240	2.5 ± 1.3	2.3 ± 1.3	255/0	240/0	187/68	171/69	60
Xu 2024	China	Retrospective	106	113	3.58 ± 2.65	3.04 ± 2.65	81/25	83/30	NA	NA	12–30
He 2025	China	Retrospective	5,244	6,054	NA		4,063/1,181	4,785/1,269	NA	NA	24
Wang 2025	China	Retrospective	60	53	NA		32/28	29/24	NA	NA	6

NA, not available; RCT, randomized controlled trial; PSM, propensity score matching; M/F, male/female.

### Quality evaluation of the included studies

The two randomized controlled trials included were evaluated based on the Cochrane Risk of Bias Assessment Tool 2 (ROB2). The results showed that the overall risk of bias in both studies was somewhat concerning (Supplementary Figure S1). Li et al. (20) were rated as low risk in the randomization process, deviation from the established intervention, and missing data, but there were some concerns in the outcome measurement domain due to the lack of blind assessment of subjective outcomes; Obata et al. (19) were rated as low risk in the randomization process and missing data, but were rated as having some concerns in the deviation from the established intervention domain due to the operation by inexperienced doctors; and due to the lack of description of blinding, the outcome measurement domain was rated as having some concerns.

The non-randomized intervention study risk of bias assessment tool (ROBINS-I) was used to evaluate the 11 non-randomized controlled studies (11, 21–30) included. The results showed that 2 studies (21, 25) using propensity score matching were rated as low risk in the dimensions of confounding factors and study subject selection, while the remaining 9 single-center retrospective studies were rated as medium risk due to insufficient correction of potential confounding factors such as history of incarceration, surgeon experience, and certain limitations in the included population; all studies were rated as low risk in the intervention measure classification, deviation from the predetermined intervention, outcome index measurement and reporting domains, except for 2 multicenter studies (22, 26) which were rated as medium risk due to the lack of follow-up data; the overall risk of bias level was low-risk for 2 studies and medium-risk for 9 studies, with no high or extremely high-risk studies (Supplementary Table S2).

### Main outcome indicators

#### Surgery time

**Figure 2:**
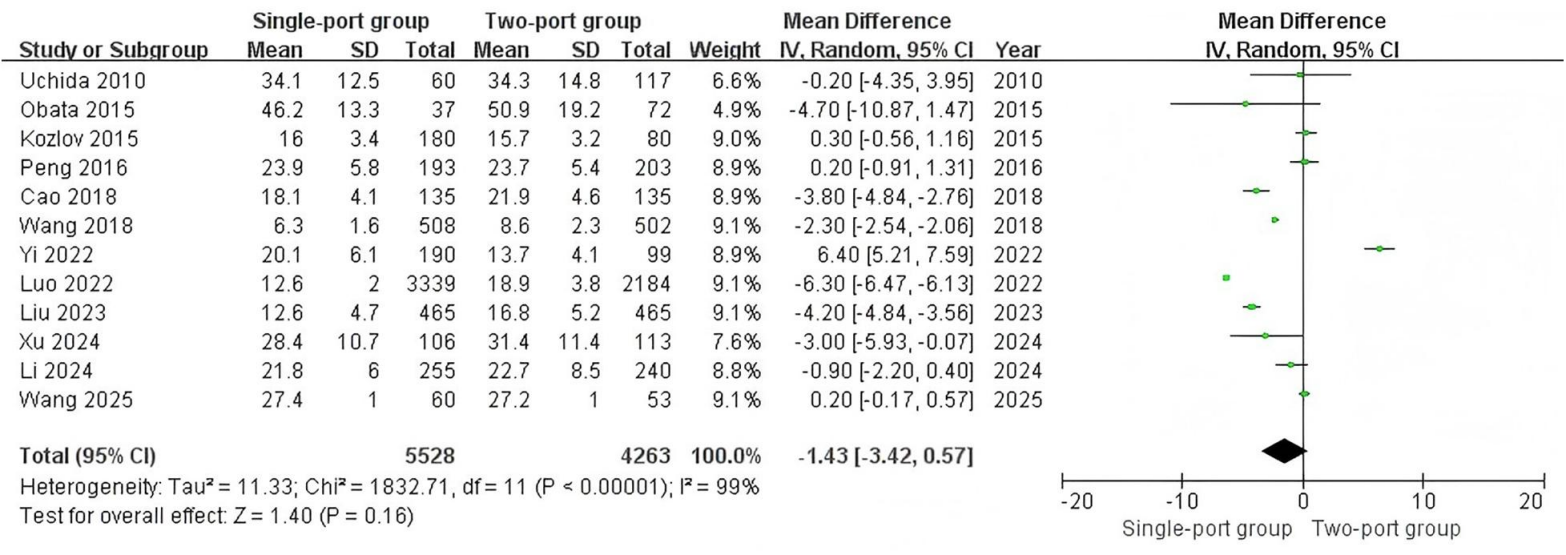
Forest plot of the meta-analysis on surgery time (minutes) for single-port and two-port.

**Figure 3:**
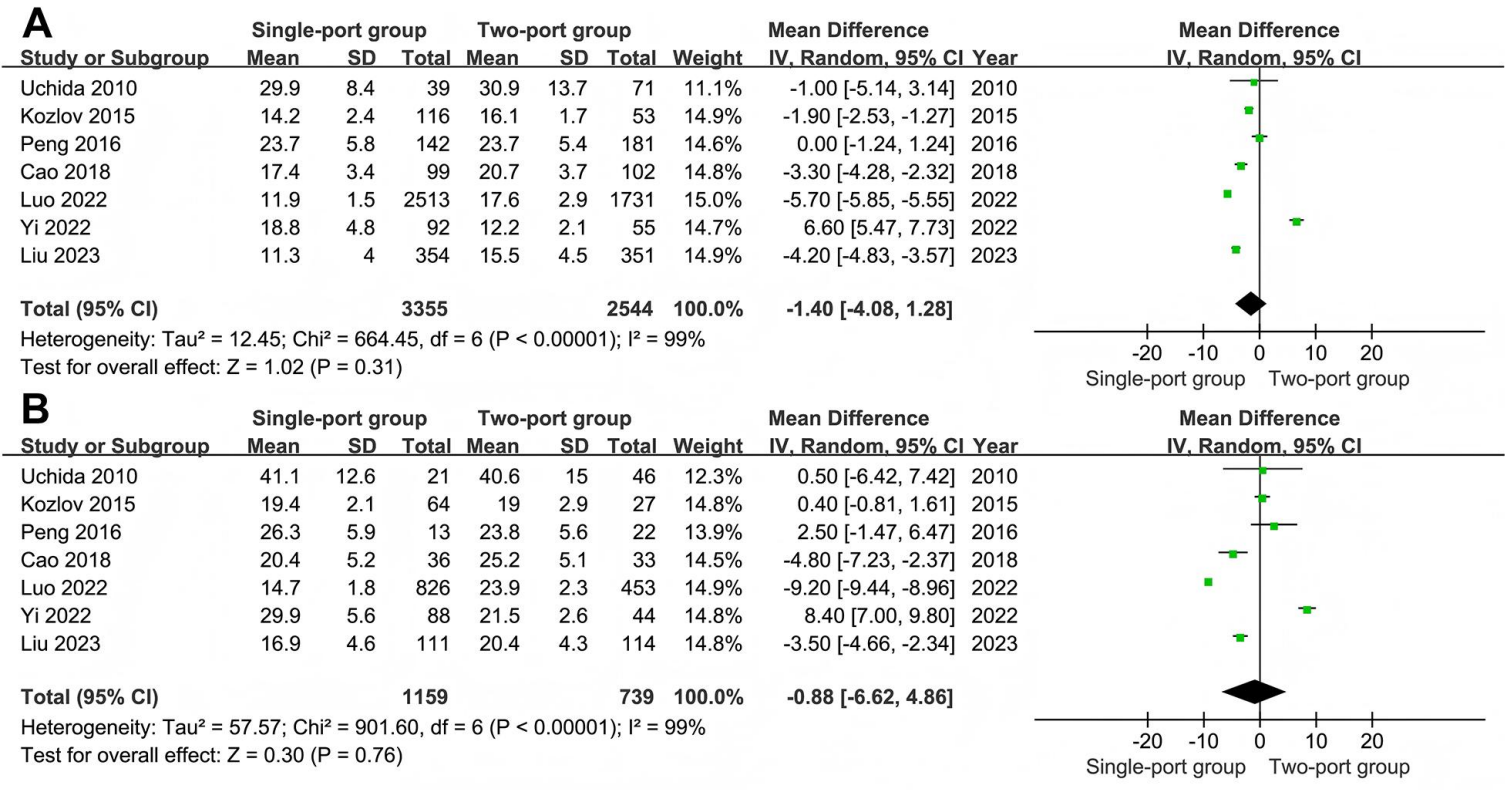
Forest plots of meta-analysis of the surgery time (minutes) for unilateral inguinal hernia **(A)** or bilateral inguinal hernia **(B)** in single-port and double-port treatments.

A total of 12 studies (11, 19–21, 23–30) reported the surgery time for both surgical approaches. The heterogeneity test showed I^2^ = 99%, indicating high heterogeneity. The meta-analysis using the random effect model revealed that there was no statistically significant difference in surgery time (MD = −1.43, 95% CI: −3.42–0.57, *P* = 0.16) (Figure 2) between the single-port group and the two-port group. Seven studies (21, 23–28) separately reported the surgery time for both surgical approaches in the treatment of unilateral inguinal hernia or bilateral inguinal hernia. The results showed that there was no significant difference in surgery time between the two surgical approaches in treating unilateral inguinal hernia (MD = −1.40, 95% CI: −4.08–1.28, *P* = 0.31) (Figure 3A) or bilateral inguinal hernia (MD = −0.88, 95% CI: −6.62–4.86, *P* = 0.76) (Figure 3B). There was high heterogeneity among the studies (I^2^ was 99% for all).

Subgroup analysis showed that after stratification by study design, there was no significant difference in surgery time between the two surgical approaches in RCT studies (MD = −1.54, 95% CI: −4.32–1.24, *P* = 0.28) and non-RCCT studies (MD = −1.29, 95% CI: −3.45–0.86, *P* = 0.24) (Supplementary Table S2). The heterogeneity in the RCT subgroup was improved (I^2^ = 27%). After stratification by sample size, there was no significant difference in surgery time between the two surgical approaches in the subgroup of small sample size studies (MD = −0.25, 95% CI: −2.01–1.52, *P* = 0.78) (Supplementary Table S3), while in the subgroup of large sample size studies (MD = −4.27, 95% CI: −7.25–1.28, *P* < 0.001) (Supplementary Table S3), the surgery time of the single-port group was significantly lower than that of the two-port group. At the same time, the heterogeneity in the two subgroups stratified by sample size was still high (I^2^ = 98% and I^2^ = 100%).

#### Recurrence rate

**Figure 4:**
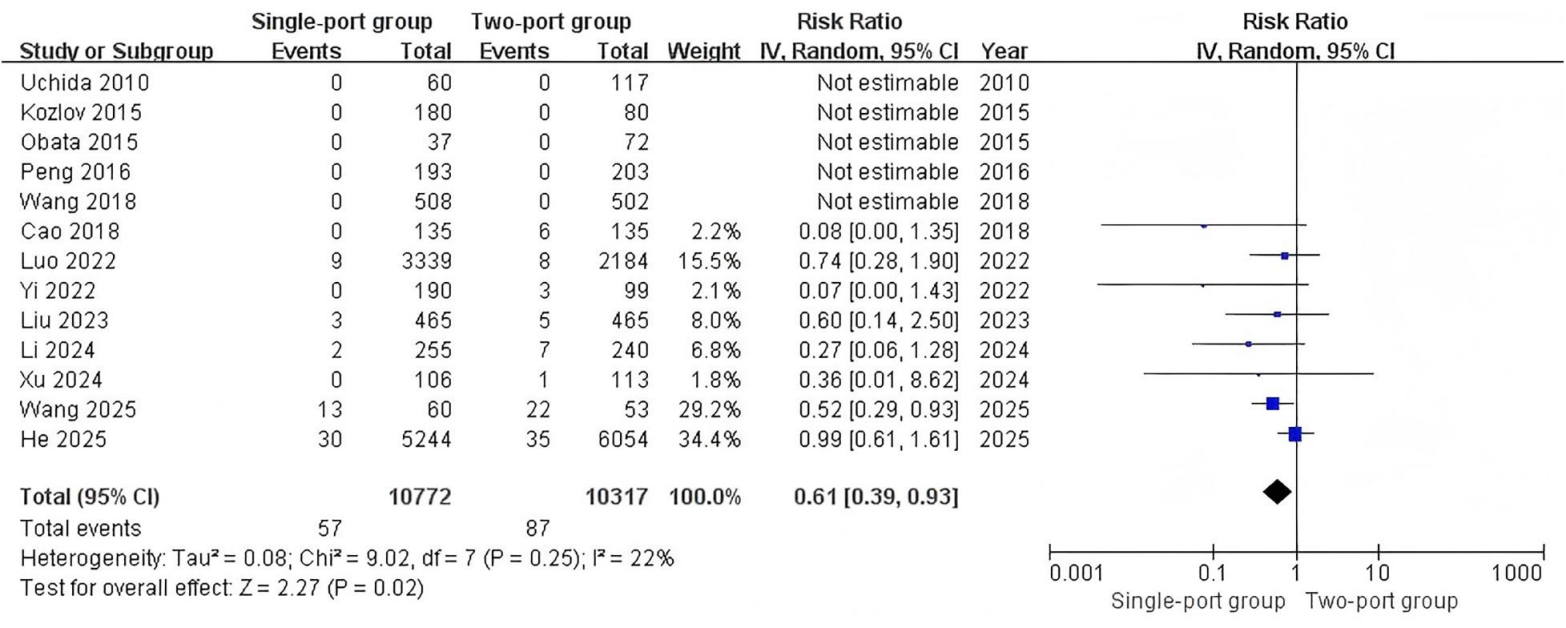
Forest plot of the meta-analysis on postoperative recurrence for single-port and two-port.

All 13 studies (11, 19–30) reported the postoperative recurrence rate. The recurrence rate in the single-port group was 0.53% (57/10,772), while that in the control group was 0.84% (87/10,317). The heterogeneity test I^2^ = 23%, indicating a low degree of heterogeneity. After using random effect meta-analysis, the results showed that the recurrence rate in the single-port group was lower than that in the two-port group (RR = 0.60, 95% CI: 0.39–0.94, *P* = 0.02) (Figure 4). Subgroup analysis revealed that after stratifying by study design, the recurrence rates of the two surgical methods in RCT studies (RR = 0.27, 95% CI: 0.06–1.28, *P* = 0.1) and non-RCT studies (RR = 0.65, 95% CI: 0.42–1.00, *P* = 0.05) (Supplementary Table S3) showed no significant difference. The heterogeneity of the RCT study subgroup improved (I^2^ = 27%). After stratifying by sample size, the recurrence rates of the two surgical methods in the large sample size study subgroup (RR = 0.90, 95% CI: 0.59–1.36, *P* = 0.61) (Supplementary Table S3) showed no significant difference, while in the small sample size study subgroup (RR = 0.43, 95% CI: 0.25–0.71, *P* = 0.001) (Supplementary Table S3), the recurrence rate in the single-port group was significantly lower than that in the two-port group. Subgroups were divided according to the follow-up time. The recurrence rates of the two surgical methods during the short follow-up time (≤12 months) (RR = 0.35, 95% CI: 0.07–1.64, *P* = 0.18) and the long follow-up time (> 12 months) (RR = 0.72, 95% CI:) (0.46–1.16, *P* = 0.18) There was no significant difference. The detailed follow-up situations of the included studies are shown in Supplementary Table S4.

#### Secondary outcome indicators

**Figure 5:**
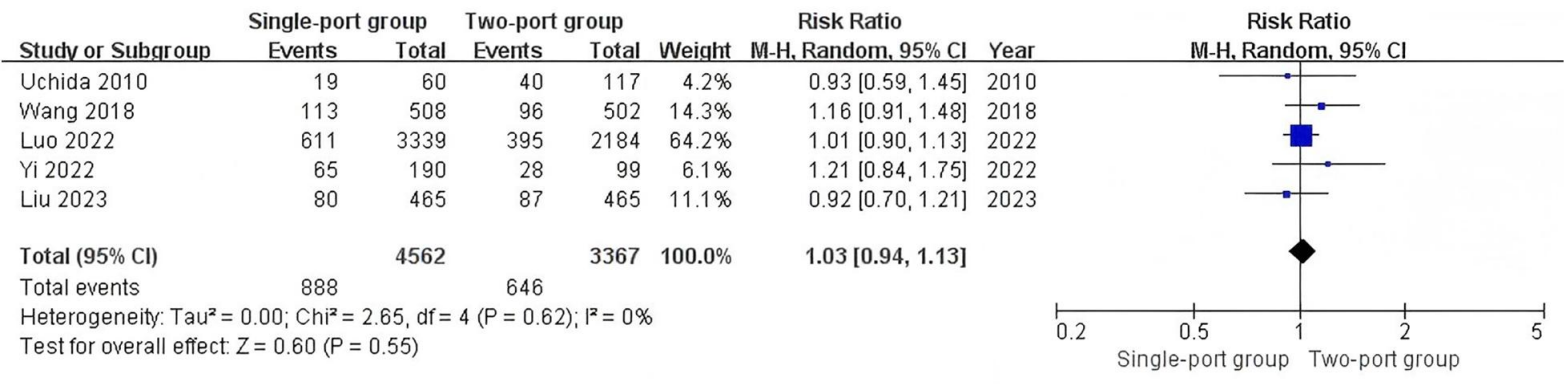
Forest plot of the meta-analysis on detection rate of contralateral hernia for single-port and two-port.

Five studies (23, 25, 26, 28, 29) reported the detection rate of contralateral hernia. The detection rate of contralateral hernia in the single-port group was 19.5% (888/4,562), and that in the two-port group was 19.2% (646/3,367). The heterogeneity test indicated low heterogeneity (I^2^ = 0%). The combined analysis results showed no significant difference in the detection rate of contralateral hernia between the two groups (RR = 1.03, 95% CI: 0.94–1.13, *P* = 0.55) (Figure 5). Twelve studies (11, 19–21, 23–30) reported the conversion to open surgery. The heterogeneity test I^2^ = 71%. The combined analysis results showed no statistical difference in the incidence of conversion to open surgery between the two groups (RR = 1.57, 95% CI: 0.14–17.93, *P* = 0.71) (Supplementary Figure S2).

Eight studies (11, 20, 21, 23, 25, 27, 29, 30) reported the incidence of surgical site infection. The heterogeneity test I^2^ = 0%. The combined analysis results showed no statistical difference in the incidence of surgical site infection between the two groups (RR = 0.58, 95% CI: 0.20–1.68, *P* = 0.32) (Table 2). Nine studies (11, 21, 23–27, 29, 30) reported the incidence of secondary hydrocele. The heterogeneity test I^2^ = 0%. The combined analysis results showed no statistical difference in the incidence of secondary hydrocele between the two groups (RR = 0.75, 95% CI: 0.42–1.34, *P* = 0.33) (Table 2). Five studies (11, 20, 21, 23, 26) reported the occurrence of suture-related complications. There was no statistical difference in the incidence of suture-related complications between the two groups (RR = 0.69, 95% CI: 0.10–4.78, *P* = 0.70) (Table 2). Four studies (23, 26, 27, 29) monitored the occurrence of bleeding or hematoma in the surgical area. The combined analysis results showed no statistical difference in the incidence of postoperative bleeding or hematoma between the two groups (RR = 0.62, 95% CI: 0.03–11.34, *P* = 0.74) (Table 2).

**Table 2:** Meta-analysis results of postoperative complications.

Postoperative complications	Study (*n*)	Heterogeneity (I^2^)	Effect size (RR)	95% CI	*P*
Infection	8	0%	0.58	0.20–1.68	0.32
Hydrocele	9	0%	0.75	0.42–1.34	0.33
Suture-related complications	5	70%	0.69	0.10–4.78	0.70
Bleeding or hematoma	4	78%	0.62	0.0.3–11.34	0.78

**Figure 6:**
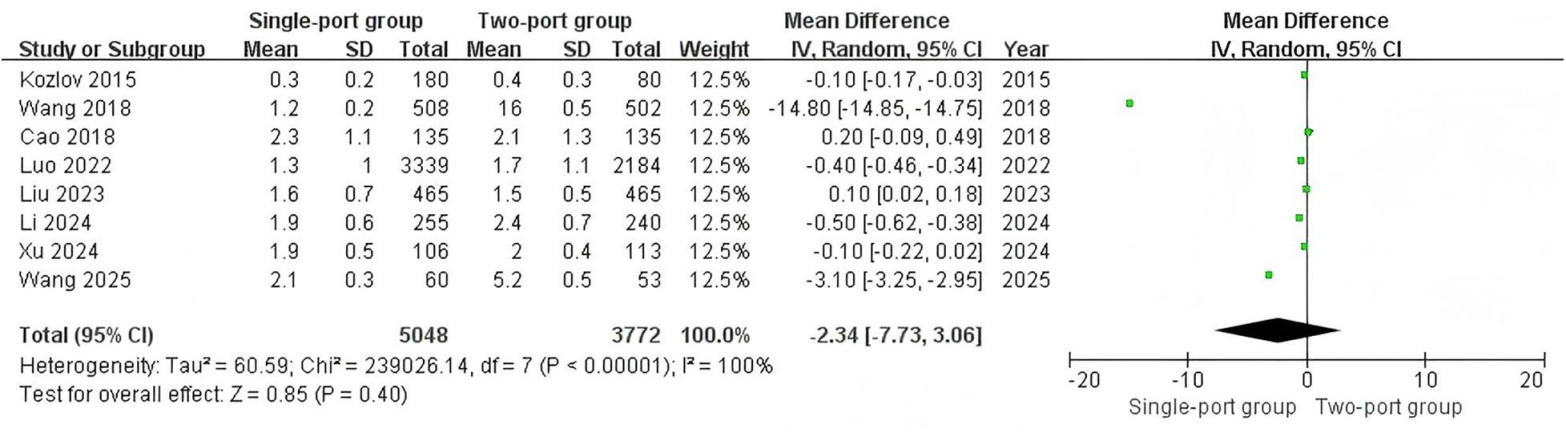
Forest plot of the meta-analysis on length of hospital stay (days) for single-port and two-port.

Eight studies (11, 20, 21, 24–26, 29, 30) reported the length of hospital stay. The heterogeneity test showed I^2^ = 99%, indicating high heterogeneity. The combined analysis results showed no significant difference in the length of hospital stay between the two surgical methods (MD = −2.34, 95% CI: −7.73–3.06, *P* = 0.40) (Figure 6).

#### Sensitivity analysis

A sensitivity analysis was conducted on the main outcome indicators. The results showed that after excluding the studies by Wang et al. (30), the combined effect size of the recurrence rate underwent a substantial change. Specifically, there was no statistically significant difference in the postoperative hernia recurrence rates between the two surgical methods (RR = 0.59, 95% CI: 0.33–1.07, *P* = 0.08) (Supplementary Figure S3); after excluding the studies by Yi et al. (23), the combined effect size of the operation time underwent a substantial change. That is, the operation time of the single-port group was lower than that of the two-port group (RR = 0.59, 95% CI: 0.33–1.07, *P* = 0.08) (Supplementary Figure S4).

#### Publication bias

**Figure 7:**
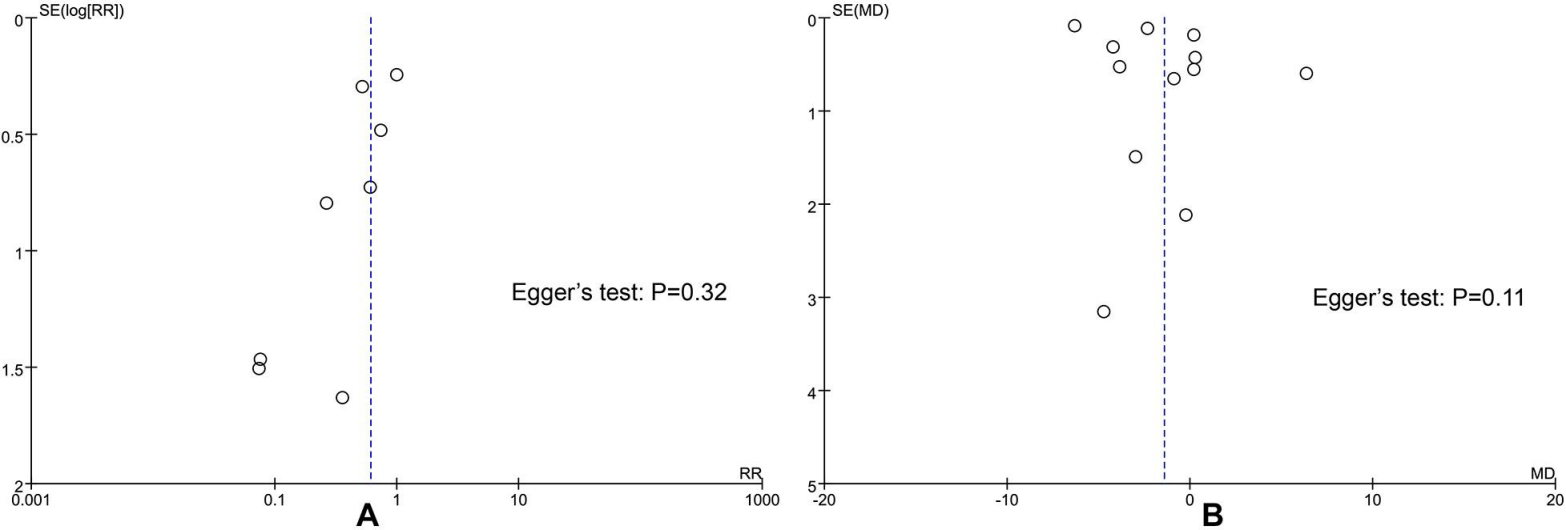
Funnel plot. **(A)** recurrence rate funnel plot; **(B)** operation time (minutes) funnel plot.

Funnel plots were drawn for the recurrence rate and operation time indicators. Visual observation showed that the funnel plots were basically symmetrical. The Egger test results indicated that there was no statistically significant difference in recurrence rate (*P* = 0.32) and operation time (*P* = 0.11) (Figure 7), suggesting that there was no obvious publication bias in the included studies.

## Discussion

This study systematically evaluated the perioperative efficacy and safety of umbilical single-port laparoscopic surgery and two-port laparoscopic surgery in the treatment of pediatric inguinal hernia. A total of 13 studies involving 22,846 children were included. The meta-analysis results showed that the postoperative recurrence rate of the single-port group was significantly lower than that of the two-port group. However, there were no significant differences in terms of operation time, hospital stay, detection rate of contralateral hernia, conversion to open surgery rate, and the incidence of various postoperative complications between the two groups. Subgroup analysis further revealed that in large-sample studies, the operation time of the single-port group was shorter, while in small-sample studies, the recurrence rate advantage of the single-port group was more significant.

The postoperative recurrence rate is the core indicator for evaluating the efficacy of hernia repair surgery. The postoperative recurrence rate of the single-port group was significantly lower than that of the multi-port group (RR = 0.60, *P* = 0.02), which was consistent with the conclusion of Wang et al. (29). The core reason might be related to the anatomical advantages of the single-port technique. The single-port laparoscopy passes through a single incision at the umbilicus to reach the surgical area, providing a more concentrated field of vision (31), enabling more precise completion of the high-position hernia sac ligation, and reducing the risk of incomplete or residual ligation (13).

In addition, consistent with the finding that single-port surgery takes less time than two-port surgery when performed by experienced surgeons, the lower recurrence rate observed in the single-port group may also be attributed to surgeon proficiency. Surgeons who can perform single-port laparoscopic procedures efficiently and safely have typically completed the full learning curve of this technique, with more precise anatomical recognition and standardized operative maneuvers, which can further reduce the risk of incomplete ligation and technical errors that lead to postoperative hernia recurrence.

However, the subgroup analysis showed that there was no significant difference in recurrence rate between the two groups in RCT studies (*P* = 0.10), which might be related to the smaller sample size and shorter follow-up time (≤2 years) of the RCT studies, while cohort studies are usually large-sample long-term follow-ups, better reflecting the true difference in recurrence rate (32). However, it is worth noting that the sensitivity analysis showed that the recurrence rate difference was not statistically significant after excluding the study by Wang 2025 et al. (30), suggesting that this result might be affected by individual studies, and the results should be interpreted with caution.

The overall analysis of this study showed no significant difference in operation time between the two groups (*P* = 0.16), but the subgroup analysis found that in large-sample studies, the operation time of the single-port group was significantly shorter (*P* < 0.001). This is consistent with the results of Fan et al. (15). Large-sample studies usually come from large medical centers, and the surgeons usually have passed the learning curve (33). This indicates that in the hands of experienced surgeons, single-port surgery not only does not prolong the operation time, but may improve efficiency by reducing the time for establishing puncture holes (26). In contrast, small-sample studies may reflect the situation in the early stage of the implementation of single-port technology or when the surgeon is inexperienced, and at this time, the operation time may be prolonged due to poor technical proficiency (19). This result suggests that the learning curve of single-port laparoscopic surgery may be a key factor affecting the operation time, and with the accumulation of the surgeon's experience, the time advantage of single-port surgery will gradually become apparent. In addition, the heterogeneity of operation time was extremely high (I^2^ = 99%) in this study, in addition to sample size differences, it may also be related to differences in the composition of hernia types, instrument types, and the standardization degree of the surgical process (34, 35). There were no significant differences in the detection rate of contralateral hernia, conversion to open surgery rate, and the incidence of various postoperative complications between the two groups, indicating that the safety of single-port and double-port laparoscopic surgery is comparable. This confirms that although single-port surgery has limited operating space, it does not increase the risk of tissue damage or infection, and the reason might be that single-port laparoscopy reduces the number of puncture holes, lowers the probability of vascular and nerve injury related to puncture, and the umbilical incision heals more covertly, with a lower risk of bacterial colonization (36, 37). There was no difference in the detection rate of contralateral indirect hernia, indicating that the abdominal cavity exploration capabilities of the two surgical methods were comparable, and both could effectively detect hidden hernias and avoid secondary surgeries, which was related to the common advantages of laparoscopic surgery (38). There was no significant difference in the conversion to open surgery rate, suggesting that even if single-port surgery encountered complex situations such as incarcerated hernia or anatomical variations, the surgeon could promptly switch the surgical method without increasing the surgical risk (39).

There was no statistical difference in the hospital stay between the two groups (MD = −2.34, *P* = 0.40), which was different from the previous research conclusion (40). With the popularization of the rapid rehabilitation surgery (ERAS) concept in pediatrics, most medical centers have established standardized postoperative management procedures (41). The postoperative fasting time, bed rest time, and discharge criteria of the two surgical methods tend to be unified, thus reducing the difference in hospital stay. In addition, the heterogeneity of hospital stay was extremely high (I^2^ = 99%), which may be related to the differences in medical resource allocation and discharge standards in different regions, such as some regions allowing discharge after 24 h of observation and some regions requiring 48–72 h of observation to ensure safety (42).

Multiple studies have shown that compared with traditional multi-port laparoscopic or open surgery, single-port surgery not only has better clinical efficacy but also brings a more ideal cosmetic effect (43, 44). For example, Chen et al. (45) confirmed that single-port laparoscopic surgery can significantly reduce postoperative scar formation and shorten recovery time, which is particularly important for pediatric patients and their families. Although single-port laparoscopic surgery has significant advantages in many clinical scenarios, its technical challenges and related risks are still key issues that clinical physicians must address. Firstly, single-port surgery relies on a single incision, limiting the surgeon's operational flexibility and field of vision, especially in cases involving complex anatomical structures or large hernias, which is more obvious (46). Compared with traditional multi-port surgery, single-port laparoscopic surgery has greater limitations in terms of operating space, instrument placement, and adjustment. This limitation is particularly prominent in handling complex hernias, such as recurrent cases or those with extensive peritoneal adhesions (47). In such cases, single-port surgery may not provide sufficient visualization or flexibility. Moreover, the learning curve associated with single-port surgery is still one of its key technical challenges. Surgeons with insufficient technical skills may face difficulties such as instrument interference and restricted working space, which may affect the surgical outcome, especially when dealing with complex anatomical structures (48). Therefore, although single-port surgery may reduce postoperative complications for pediatric inguinal hernia, its technical complexity and the professional level of surgeons are still key factors affecting success (49).

The key advantage of this meta-analysis lies in its comprehensive review of the first comparison between single-port and two-port laparoscopic techniques for the treatment of pediatric inguinal hernias. Secondly, sufficient subgroup analysis and sensitivity analysis were conducted to explore the sources of heterogeneity and verify the robustness of the results. Moreover, the included studies evaluated various outcome indicators, such as recurrence rate, operation duration, and complication rate, thereby providing a comprehensive evaluation of the performance of each technique. However, this study also has several limitations. The significant differences in design and sample size among the included studies may lead to bias and affect the generalizability of the results. Secondly, the follow-up period of some studies was relatively short, and the recurrence rate might increase with the extension of the follow-up period, which could be an important potential confounding factor. Additionally, some outcome indicators, such as operation time and hospital stay, have high heterogeneity. Although a random effects model was used, the combined results should be interpreted with caution; finally, differences in surgeons' experience, follow-up time, etc. among different studies may affect the consistency of the results.

In the future, more large-scale, multi-center, and high-quality RCTs should be conducted to further verify the long-term efficacy of single-port laparoscopic surgery; explore the optimal indications for single-port surgery, such as differences in efficacy among different age groups and different types of hernias (incarcerated hernia, recurrent hernia) in children. At the same time, cost-effectiveness analysis should be carried out to compare the economic characteristics of the two surgical methods.

In conclusion, the results of this study indicate that umbilical single-port laparoscopic surgery for pediatric inguinal hernia has a significant advantage in reducing postoperative recurrence rate. It demonstrates a surgical time advantage in large sample sizes and experienced medical centers, and its safety is comparable to two-port laparoscopic surgery. For medical centers with mature single-port operation techniques, single-port laparoscopic surgery can be the preferred approach; for complex hernia cases or medical units with limited technical conditions, two-port laparoscopic surgery remains a reliable option.

## Supplementary material

SUPPLEMENTARY FIGURE 1 Results of the risk of bias assessment using RoB2.

SUPPLEMENTARY FIGURE 2 Forest plot of the meta-analysis on conversion to open surgery for single-port and two-port.

SUPPLEMENTARY FIGURE 3 Forest plot of postoperative hernia recurrence after sensitivity analysis.

SUPPLEMENTARY FIGURE 4 Forest plot of operation time (days) after sensitivity analysis.
