# Supplementary figures and images for: Efficacy of transumbilical single-port and two-port laparoscopy in the treatment of pediatric inguinal hernia: a systematic review and meta-analysis
Source: Front Pediatr. 2026 May 8;14:1814850. doi: 10.3389/fped.2026.1814850 (PMC13194568; doi:10.3389/fped.2026.1814850)

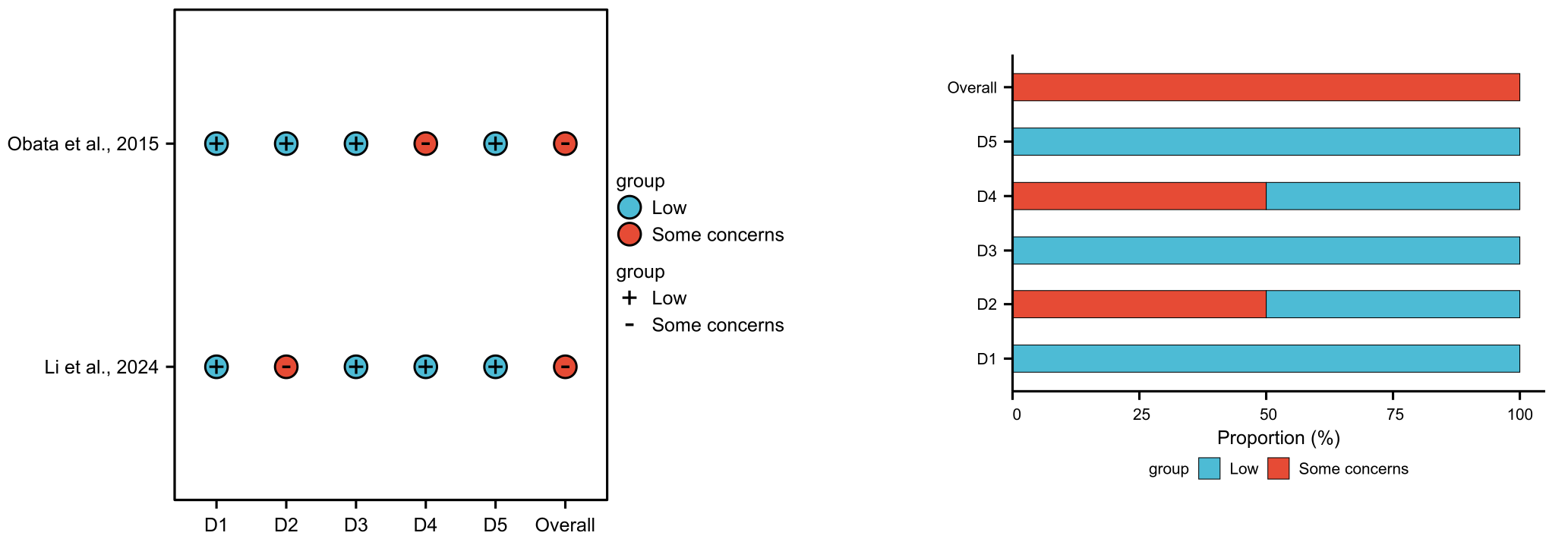

Supplement: SUPPLEMENTARY FIGURE 1 — Results of the risk of bias assessment using RoB2. [file Image1.png]

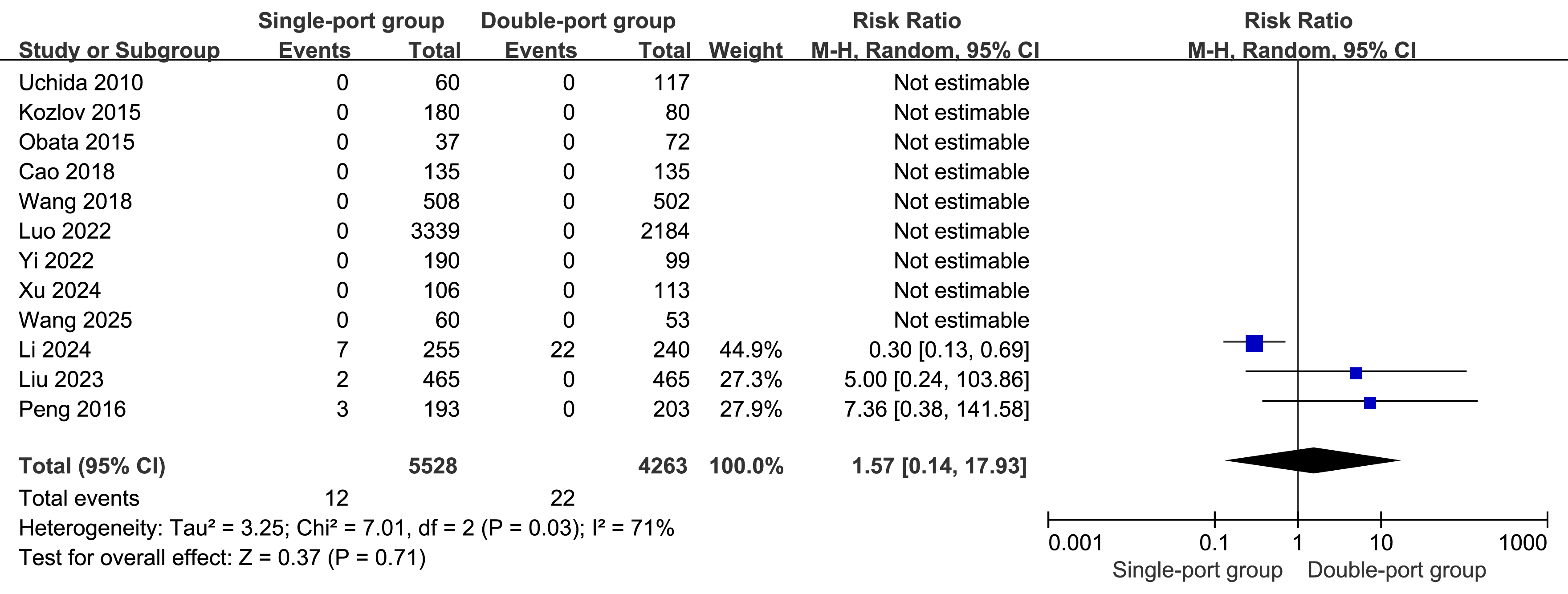

Supplement: SUPPLEMENTARY FIGURE 2 — Forest plot of the meta-analysis on conversion to open surgery for single-port and two-port. [file Image2.png]

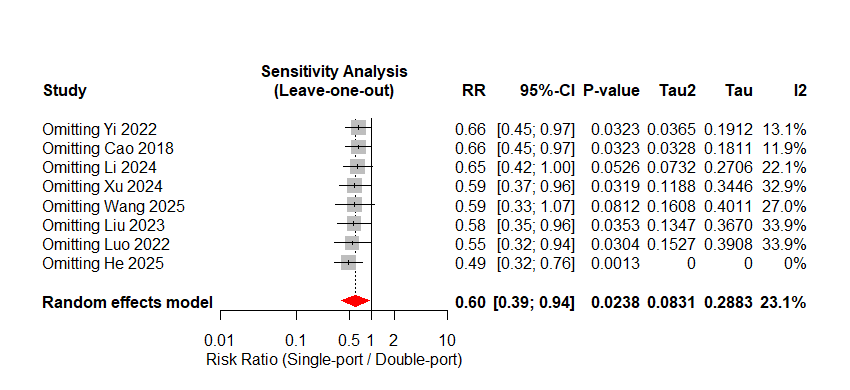

Supplement: SUPPLEMENTARY FIGURE 3 — Forest plot of postoperative hernia recurrence after sensitivity analysis. [file Image3.png]

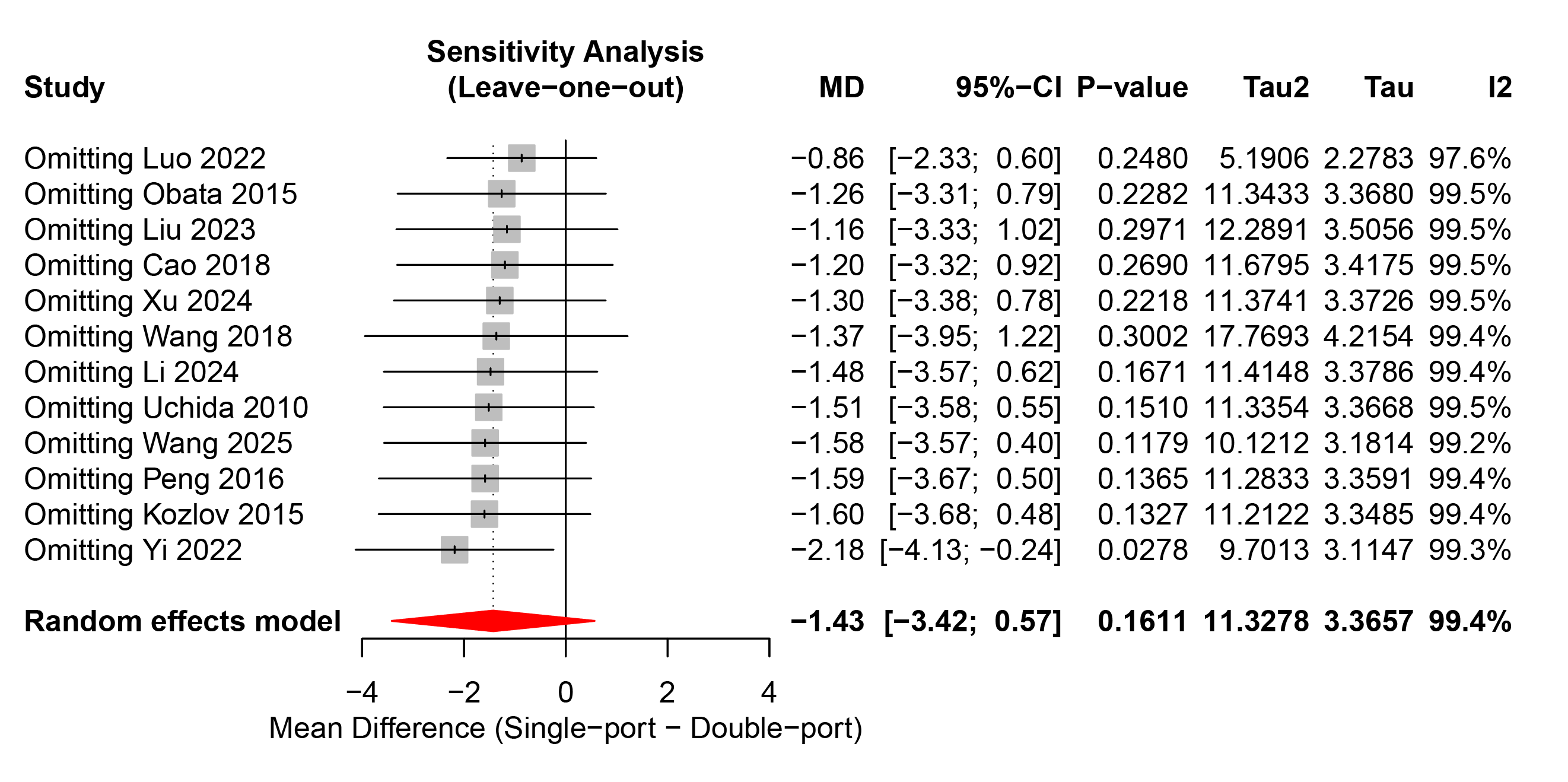

Supplement: SUPPLEMENTARY FIGURE 4 — Forest plot of operation time (days) after sensitivity analysis. [file Image4.png]
