# Supplementary material for: Efficacy of transumbilical single-port and two-port laparoscopy in the treatment of pediatric inguinal hernia: a systematic review and meta-analysis
Source: Front Pediatr. 2026 May 8;14:1814850. doi: 10.3389/fped.2026.1814850 (PMC13194568; doi:10.3389/fped.2026.1814850)
Supplement: Supplementary file 5 [file Table1.docx]

Supplemental Table S1. Detailed search strategies for each database.

| Database | Search strategy |
| --- | --- |
| PubMed | (("Inguinal hernia"[MeSH Terms] OR "Congenital inguinal hernia"[Title/Abstract] OR "Indirect inguinal hernia"[Title/Abstract]))  AND  (("Pediatrics"[MeSH Terms] OR "Pediatric"[Title/Abstract] OR "Paediatric"[Title/Abstract] OR "Children"[Title/Abstract] OR "Infant"[Title/Abstract] OR "Adolescent"[Title/Abstract]))  AND  (("Laparoscopy"[MeSH Terms] OR "Laparoscopic surgery"[Title/Abstract] OR "Laparoscopic repair"[Title/Abstract] OR "Laparoscopic herniorrhaphy"[Title/Abstract]))  AND  (("Single-port laparoscopic"[Title/Abstract] OR "Single-incision laparoscopic"[Title/Abstract] OR "Transumbilical single-port laparoscopic"[Title/Abstract] OR "Two-port laparoscopic"[Title/Abstract] OR "Dual-port laparoscopic"[Title/Abstract] OR "Multiport laparoscopic"[Title/Abstract])) |
| Embase | (("inguinal hernia"[Emtree] OR "congenital inguinal hernia"[Title/Abstract] OR "indirect inguinal hernia"[Title/Abstract]))  AND  (("pediatric"[Emtree] OR "paediatric"[Title/Abstract] OR "children"[Title/Abstract] OR "infant"[Title/Abstract] OR "adolescent"[Title/Abstract]))  AND  (("laparoscopy"[Emtree] OR "laparoscopic surgery"[Title/Abstract] OR "laparoscopic repair"[Title/Abstract] OR "laparoscopic herniorrhaphy"[Title/Abstract]))  AND  (("single-port laparoscopic"[Title/Abstract] OR "single-incision laparoscopic"[Title/Abstract] OR "single-site laparoscopic"[Title/Abstract] OR "transumbilical single-port laparoscopic"[Title/Abstract] OR "two-port laparoscopic"[Title/Abstract] OR "dual-port laparoscopic"[Title/Abstract] OR "multiport laparoscopic"[Title/Abstract])) |
| Cochrane Library | (("inguinal hernia"[MeSH Terms] OR "congenital inguinal hernia"[All Fields] OR "indirect inguinal hernia"[All Fields]))  AND  (("pediatric"[MeSH Terms] OR "paediatric"[All Fields] OR "children"[All Fields] OR "infant"[All Fields] OR "adolescent"[All Fields]))  AND  (("laparoscopy"[MeSH Terms] OR "laparoscopic surgery"[All Fields] OR "laparoscopic repair"[All Fields] OR "laparoscopic herniorrhaphy"[All Fields]))  AND  (("single-port laparoscopic"[All Fields] OR "single-incision laparoscopic"[All Fields] OR "transumbilical single-port laparoscopic"[All Fields] OR "two-port laparoscopic"[All Fields] OR "dual-port laparoscopic"[All Fields] OR "multiport laparoscopic"[All Fields])) |
| Web of Science | TS=(("inguinal hernia" OR "congenital inguinal hernia" OR "indirect inguinal hernia"))  AND TS=(("pediatric" OR "paediatric" OR "children" OR "infant" OR "adolescent"))  AND TS=(("laparoscopy" OR "laparoscopic surgery" OR "laparoscopic repair" OR "laparoscopic herniorrhaphy"))  AND TS=(("single-port laparoscopic" OR "single-incision laparoscopic" OR "transumbilical single-port laparoscopic" OR "two-port laparoscopic" OR "dual-port laparoscopic" OR "multiport laparoscopic")) |
