# Supplementary material for: Efficacy of transumbilical single-port and two-port laparoscopy in the treatment of pediatric inguinal hernia: a systematic review and meta-analysis
Source: Front Pediatr. 2026 May 8;14:1814850. doi: 10.3389/fped.2026.1814850 (PMC13194568; doi:10.3389/fped.2026.1814850)
Supplement: Supplementary file 6 [file Table2.docx]

| Supplementary Table S2. Risk of bias in included studies assessed using the ROBINS-I tool | | | | | | | | | |
| --- | --- | --- | --- | --- | --- | --- | --- | --- | --- |
| Author | Year | Type of bias | | | | | | | Overall rating |
|  |  | Confounding | Selection of participants | Exposure assessment | Misclassification during follow-up | Missing data | Measurement of the outcome | Selective reporting of the results |  |
| Uchida | 2013 | Moderate | Moderate | Low | Low | Low | Low | Low | Moderate |
| Kozlov 2015 | 2014 | Moderate | Moderate | Low | Low | Low | Low | Low | Moderate |
| Peng 2016 | 2016 | Moderate | Moderate | Low | Low | Low | Low | Low | Moderate |
| Cao 2018 | 2016 | Low | Low | Low | Low | Low | Low | Low | Low |
| Wang 2018 | 2016 | Moderate | Moderate | Low | Low | Low | Low | Low | Moderate |
| Luo2022 | 2018 | Moderate | Moderate | Low | Low | Moderate | Low | Low | Moderate |
| Yi 2022 | 2019 | Moderate | Moderate | Low | Low | Low | Low | Low | Moderate |
| Liu 2023 | 2020 | Low | Low | Low | Low | Low | Low | Low | Low |
| Xu 2024 | 2020 | Moderate | Moderate | Low | Low | Low | Low | Low | Moderate |
| He 2025 | 2021 | Moderate | Moderate | Low | Low | Moderate | Low | Low | Moderate |
| Wang 2025 | 2021 | Moderate | Moderate | Low | Low | Low | Low | Low | Moderate |
