# Supplementary material for: Efficacy of transumbilical single-port and two-port laparoscopy in the treatment of pediatric inguinal hernia: a systematic review and meta-analysis
Source: Front Pediatr. 2026 May 8;14:1814850. doi: 10.3389/fped.2026.1814850 (PMC13194568; doi:10.3389/fped.2026.1814850)
Supplement: Supplementary file 7 [file Table3.docx]

| Supplementary Table S3. Summary Table of Subgroup Analysis Results | | | | | | |
| --- | --- | --- | --- | --- | --- | --- |
| Primary Outcomes​​ | Subgroup | Number | MD or RR | 95%CI (lower, upper) | I^2^ | P-value |
| Operation time | Study design |  |  |  |  |  |
|  | N-RCT | 10 | -1.29 | (-3.45, 0.86) | 100% | 0.24 |
|  | RCT | 2 | -1.56 | (-4.37, 1.26) | 28% | 0.28 |
|  | Sample size |  |  |  |  |  |
|  | ˃ 500 | 3 | -4.27 | (-7.25, 1.28) | 100% | ˂ 0.001 |
|  | ≤ 500 | 9 | -0.25 | (-2.01, 1.52) | 95% | 0.78 |
| Recurrence rate | Study design |  |  |  |  |  |
|  | N-RCT | 11 | 0.65 | (0.42, 1.00) | 22% | 0.05 |
|  | RCT | 2 | 0.27 | (0.06, 1.28) | NA | 0.10 |
|  | Sample size | | | | | |
|  | ˃ 500 | 4 | 0.90 | (0.59, 1.36) | 0% | 0.61 |
|  | ≤ 500 | 9 | 0.43 | (0.25, 0.71) | 0% | 0.001 |
|  | Follow-up time | | | | | |
|  | ˃ 12 | 8 | 0.72 | (0.46,1.16) | 11% | 0.18 |
|  | ≤ 12 | 5 | 0.35 | (0.07,1.64) | 38% | 0.18 |
