# Supplementary material for: Efficacy of transumbilical single-port and two-port laparoscopy in the treatment of pediatric inguinal hernia: a systematic review and meta-analysis
Source: Front Pediatr. 2026 May 8;14:1814850. doi: 10.3389/fped.2026.1814850 (PMC13194568; doi:10.3389/fped.2026.1814850)
Supplement: Supplementary file 8 [file Table4.docx]

Supplementary Table S4. Detailed Information on the Follow-up Periods of the Included Studies

| Study |  |
| --- | --- |
| Uchida 2010 | **Planned Follow-up Time:** All patients were scheduled for routine follow-up visits at 1 week​ and 3 months​ after surgery.  **Actual Reported Follow-up Time:** Conventional LPEC group: 6.5 ± 1.8 months. Single-incision SILPEC group: 1.4 ± 1.2 months.  **Subgroup:** ≤ 12 months |
| Kozlov 2015 | **Planned Follow-up Time:** Postoperative complications were monitored during the hospital stay. Furthermore, repeated checkups were scheduled at 1, 3, 6, and 12 months​ after discharge from the hospital.  **Actual Reported Follow-up Time:** The study states that patient follow-up was conducted for at least 6 months​ after surgery.  **Subgroup:** ≤ 12 months |
| Obata 2015 | **Planned Follow-up Time:** All patients were scheduled for routine follow-up visits at 1 week, 1 month, 3 months, 6 months, and 1 year​ after being discharged from the hospital.  **Actual Reported Follow-up Time:** The study results, including the analysis of complications and recurrence, were reported based on data collected during the follow-up period (1 week to 1 year after surgery).  **Subgroup:** ≤ 12 months |
| Peng 2016 | **Planned Follow-up Time:** The follow-up visits for the children were scheduled at 1, 3, 6, and 12 months postoperatively, and annually thereafter.  **Actual Reported Follow-up** Time: Of the 396 children in the study, 393 children (99.2%) completed at least 36 months of follow-up. The follow-up period for these patients ranged from 36 to 108 months, with a mean follow-up time of 48.2 months. Three children were lost to follow-up.  **Subgroup:** >12 months |
| Cao 2018 | **Planned Follow-up Time:** Patients were discharged and scheduled for follow-up visits in the outpatient clinic at 2 weeks, 1 month, 3 months, 6 months, 1 year, and 2 years​ after the operation.  **Actual Reported Follow-up Time:** The study states that the follow-up lasted from 1 month to 2 years. Outcomes, including recurrence rates, were reported based on this period. Specifically, the results mention that there were "no hernia recurrences noted in boys within 2 years postoperatively" and that the cosmetic results were assessed "based on the follow-up of >6 months". Therefore, the actual follow-up period for analysis spanned at least from 1 month to 2 years post-surgery.  **Subgroup:** >12 months |
| Wang 2018 | **Planned Follow-up Time:** The study does not explicitly detail a scheduled follow-up protocol.  **Actual Reported Follow-up Time:** The study reports that all children (n=1,010) were followed up for 2 years. Key outcomes, including hernia recurrence (0 in the single-site group vs. 2 in the double-site group) and the appearance of contralateral occult hernias, were assessed and reported based on this 2-year follow-up period.  **Subgroup:** >12 months |
| Luo2022 | **Planned Follow-up Time:** Routine outpatient follow-up visits were scheduled for all patients at 1, 3, 6, and 12 months​ after discharge from the hospital.  **Actual Reported Follow-up Time:** All enrolled patients (n=5523) were successfully followed up. The follow-up period for all patients ranged from 12 to 93 months, with a mean follow-up time of 54 months.  **Subgroup:** >12 months |
| Yi 2022 | **Planned Follow-up Time:** The study does not explicitly detail a scheduled follow-up protocol.  **Actual Reported Follow-up Time:** The short-term outcomes were reported based on a mean follow-up duration of 9.8 ± 3.4 months​ for the TUSLIC group and 9.6 ± 3.2 months​ for the TAMLEC group, with no statistically significant difference between the groups (P=0.81). The authors note in the discussion that a limitation of the study is its relatively short follow-up period (mean about 9.5 months).  **Subgroup:** ≤ 12 months |
| Liu 2023 | **Planned Follow-up Time:** The study protocol required a minimum follow-up period of 6 months​ for inclusion in the analysis. Patients with a follow-up time of less than 6 months were excluded from the study.  **Actual Reported Follow-up Time:** After propensity score matching, the median follow-up times for the two patient groups were: SLPEC (Single-port) group: 32.15 ± 7.33 months. TLPEC (Two-port) group: 32.30 ± 8.97 months.  **Subgroup:** >12 months |
| Li 2024 | **Planned Follow-up Time:** The study protocol scheduled outpatient follow-up interviews for all patients at 2 weeks, 3 months, 1 year, 3 years, and 5 years​ after the surgical intervention.  **Actual Reported Follow-up Time:** The study was specifically designed to analyze follow-up data over 5 years. The primary and secondary outcomes, including recurrence rates and complications, were reported based on this 5-year follow-up period. The results section explicitly states analyses such as "During the 5-year follow-up period" and primary outcome analysis "at 5-year follow-up."  **Subgroup:** >12 months |
| Xu 2024 | **Planned Follow-up Time:** The study does not explicitly detail a scheduled follow-up protocol.  **Actual Reported Follow-up Time:** The study reports a mean follow-up duration of 19.81 ± 6.03 months (range: 12.00-30.00 months)​ for the SLPEC group and 18.40 ± 5.58 months (range: 12.00-30.50 months)​ for the TLPEC group.  **Subgroup:** >12 months |
| He 2025 | **Planned Follow-up Time:** The postoperative follow-up period for all patients in the study was planned to be 2 years. Follow-up assessments were specifically scheduled at 1 month, 3 months, and 2 years​ after surgery.  **Actual Reported Follow-up Time:** The study explicitly states that all patients were followed for 2 years. The analysis of recurrence rates and the intervals to recurrence (ranging from 0 to 60 months) are based on data collected during this 2-year follow-up period.  **Subgroup:** >12 months |
| Wang 2025 | **Planned Follow-up Time:** The study protocol specified that a 6-month follow-up​ was conducted to evaluate and compare recurrence and reoperation rates between the two surgical groups.  **Actual Reported Follow-up Time:** The study results, including the analysis of recurrence rates and reoperation rates, are explicitly reported based on data collected from this 6-month follow-up period.  **Subgroup:** ≤ 12 months |
